# Assessing the impact of adjuvant therapy on cure rate for stage 2 breast carcinoma.

**DOI:** 10.1038/bjc.1993.296

**Published:** 1993-07

**Authors:** J. W. Gamel, R. L. Vogel, I. W. McLean

**Affiliations:** Veterans Administration Medical Center, Louisville, KY.

## Abstract

The log-rank test is commonly used to assess therapeutic effect in prospective, randomised clinical trials. This test is sensitive to differences in survival between treatment groups at a specific endpoint, but cannot determine whether such a difference is due to an enhanced cure rate or an enhanced survival time among uncured patients. To investigate the clinical impact of such limitations, an algorithm was constructed to simulate clinical, randomised, adjuvant therapy trials in patients with a cured fraction of 0.27 and a median survival time for uncured patients of 3.4 years. Hypothetical therapies were introduced to increase rate of cure, increase median survival time, or achieve a combination of these effects. For 500 simulated patients recruited over a 5 year period and then followed for three additional years, a 50% enhancement of median survival time (to 5.1 years) led to a survival increase detectable at the P = 0.05 level in 780 of 1000 trials, whereas a 50% enhancement of cured fraction (to 40.5%) led to a detectable increase at the same level in only 449 of 1000 trials. These findings suggest that, in clinical trials of adjuvant therapy for stage 2 breast cancer, the log rank test may be more sensitive to increases in tumour-related survival time than to increases in cured fraction.


### Assessing the impact of adjuvant therapy on cure rate for stage 2 breast carcinoma

J.W. Gamel[1], R.L. Vogel[2] & I.W. McLean[3]

[1]*Veterans Administration Medical Center and University of Louisville School of Medicine, Department of Ophthalmology and Visual Sciences, 301 E. Muhammad Ali Blvd., Louisville, KY 40292;* [2]*University of Louisville, Department of Computing and Telecommunications, Louisville, KT 40292; and* [3]*Armed Forces Institute of Pathology, Department of Ophthalmic Pathology, Washington, DC 20306, USA.*

**Summary** The log-rank test is commonly used to assess therapeutic effect in prospective, randomised clinical trials. This test is sensitive to differences in survival between treatment groups at a specific endpoint, but cannot determine whether such a difference is due to an enhanced cure rate or an enhanced survival time among uncured patients. To investigate the clinical impact of such limitations, an algorithm was constructed to simulate clinical, randomised, adjuvant therapy trials in patients with a cured fraction of 0.27 and a median survival time for uncured patients of 3.4 years. Hypothetical therapies were introduced to increase rate of cure, increase median survival time, or achieve a combination of these effects. For 500 simulated patients recruited over a 5 year period and then followed for three additional years, a 50% enhancement of median survival time (to 5.1 years) led to a survival increase detectable at the $P = 0.05$ level in 780 of 1000 trials, whereas a 50% enhancement of cured fraction (to 40.5%) led to a detectable increase at the same level in only 449 of 1000 trials. These findings suggest that, in clinical trials of adjuvant therapy for stage 2 breast cancer, the log rank test may be more sensitive to increases in tumour-related survival time than to increases in cured fraction.

Randomised clinical trials of adjuvant therapy are designed to detect a difference in survival rates than can be attributed to the therapy under investigation. Although this difference in survival is a paradigm of modern clinical trials, it provides little insight into the biological processes responsible for the change in survival rate.

At the biological level, cancer therapy can enhance survival at a specific end-point by two distinct mechanisms: an increase in cured fraction, and a lengthening of survival times among uncured patients (Boag, 1949; Gamel *et al.*,1990; Mould & Boag, 1975). Ideally, we would like to know the impact of therapy on each of these mechanisms. To make such a determination from survival analysis alone, however, we would need more patients and more follow up than are feasible for most clinical trials, as can be seen in Figure 1. Figure 1a shows the impact of a 50% increase in cured fraction for a hypothetical population of patients with stage 2 breast carcinoma, while Figure 1b demonstrates the impact on this population of a 50% increase in median survival time. Within the 5–10 year limit of most clinical trials, confidence intervals are too wide to allow a clear distinction between these two survival patterns. Thus, even if a clinical trial yields a significant difference in survival at the end of a specified time interval, we may remain uncertain whether this finding reflects a higher rate of cure, a prolongation of survival time, of a combination of these mechanisms.

An important step in designing a clinical trial is estimation of the sample size needed to have a reasonable chance of detecting clinically significant differences in survival rates. The likelihood of observing a statistically significant difference in survival is referred to as the power of the trial, and there are published tables that relate sample size, magnitude of the difference to be detected, level of statistical significance, and power desired (Freedman, 1982). For example, a clinical trial is conducted in which patients in the control group have a 30% survival rate. If the treatment under study results in an improvement in survival to 45%, then a sample size of 307 patients is required so that 80% of such studies will have differences in survival significant at the

**Figure 1** The continuous line represents predicated survival for a hypothetical population of patients with cured fraction of 0.27, median survival time of 3.4 years, standard deviation log survival time of 1.04, and a lognormal distribution of time to death from tumour. These parameters are the same as those found by Rutqvist for a population of 5252 patients with stage 2 breast cancer. **a**, Upper broken line represents predicted survival for this population, assuming a 50% increase in cured fraction from treatment. Lower broken line represents the difference in survival between treated and untreated patients. **b**, Upper broken line represents predicted survival for this population, assuming a 50% increase in median survival time from treatment. Lower broken line represents the difference in survival between treated and untreated patients.

Correspondence: Dr John W. Gamel, Department of Ophthalmology & Visual Sciences, 301 E Muhammad Ali Blvd., Louisville, KY, 40292, USA.
Received 30 September 1992; and in revised form 10 February 1993.

level of $P = 0.05$. To increase the power of this trial from 80 to 90%, the sample size would have to be increased to 411 patients (Freedman, 1982).

It is important to note that these sample-size estimates were derived from standard tables, which are predicted entirely on survival differences at a specific endpoint. Thus these tables offer no insight into the relative impact of two important parameters – cured fraction and median survival time. Furthermore, there is no specific allowance for interaction of these parameters with clinical covariates, such as age of the patient. To overcome such limitations, we have devised an algorithm that simulates the dynamics of patients treated with adjuvant therapy more closely than is possible with standard tables.

Using this algorithm, we will address the power of a clinical trial for stage 2 breast carcinoma from a different perspective: Given a treatment that achieves biologically meaningful improvements in cure rate, median survival time, or both, how does our ability to detect a statistically significant survival difference vary as a function of number of patients enrolled and duration of the study?

### Materials and methods

### *The lognormal survival model*

Early and important insight into mortality from cancer was gained with the classic publication of Boag in 1949. In this landmark article, he distinguished the role of cured fraction from that of time to death among uncured patients. He also showed that the distribution of time to death from many cancers was closely approximated by a lognormal function.

Expanding upon this initial work, Rutqvist studied 14,731 patients with breast carcinoma, including a sub-population of 5.252 patients with stage 2 disease (Rutqvist *et al.*, 1984). He confirmed the lognormal function as a good fit to the distribution of time to death from breast carcinoma, and characterised the distribution of age among patients with each stage of breast cancer. Furthermore, he found that cured fraction varies substantially as a function of both patient age and tumour stage, while median survival time varies substantially with tumour stage but only minimally with patient age. For stage 2 patients, the overall cured fraction was 0.27 and the median survival time was 3.4 years.

### *The population algorithm*

This algorithm was designed to generate data sets for survival analysis of patients with stage 2 breast carcinoma. In these sets, survival-related covariates followed essentially the same distributions found by Rutqvist in his sub population of 5252 patients. To achieve such distributions, patients were randomly assigned ages that followed a Gaussian distribution, with a mean of 55.2 years and a standard deviation of 12.9 years, omitting values less than 10 years or greater than 90 years. The following regressions were used to achieve a relationship of age to cured fraction and mean and standard deviation of log survival time similar to that found by Rutqvist:

$C =$ Cured fraction $= 0.97 - 0.0077$ Age
$M =$ Mean log survival time $= 1.42 + 0.011$ Age
$S =$ SD log survival time $= 0.80 + 0.0084$ Age

Therapeutic effect was determined by first randomly assigning each patient to the control group ($T = 0$) or the treatment group ($T = 1$). For treated patients, there was an enhancement of cured fraction, an enhancement of median survival time, or a combination of these effects:

$\Delta_C$ = Proportional therapeutic enhancement of cured fraction
$= 0, 0.1, 0.2, \ldots 0.9, 1.0$
$\Delta_M$ = Proportional therapeautic enhancement of median survival time
$= 0, 0.1, 0.2, \ldots 0.9, 1.0$
$C'$ = Cured fraction after treatment
$= C\ (1 + T\ \Delta_C)$
$M'$ = Mean log survival time after treatment
$= M + \log\ \{1 + T\ \Delta_M\}$

To determine whether a patient was dead of tumour; a random number uniformly distributed between 0 and 1 was selected. If this number was greater than $C'$, the patient was considered dead of tumour at a time randomly selected from a lognormal distribution with mean $M'$ and standard deviation S. Uniformly distributed random numbers were generated by the HP9836 BASIC algorithm, while those from a Gaussian distribution were generated by the Box-Muller algorithm (Hewlett-Packard, 1985; Morgan, 1984).

Note that for $T = 0$ or for $\Delta_C = \Delta_M = 0$, there is no change in cured fraction (i.e., $C' = C$) or median survival time (i.e., $\exp\{M'\} = \exp\{M\}$), while for $T = \Delta_C = \Delta_M = 1$, both parameters are increased by a factor of 2 for treated patients (i.e., $T = 1$, $C' = 2\ C$, $\exp\{M'\} = 2\ \exp\{M\}$). For $\Delta_C = \Delta_M = 0.5$, both parameters are enhanced by 50 percent for treated over untreated patients.

Allowance was also made for death from causes other than breast carcinoma. Each patient was randomly assigned an integer between 1 and 9. An examination was then made of standardised survival data for the general population of females in the United States for 1980. If, for example, a hypothetical patient was assigned an age of 55 years and an integer of 7, then this patient was assigned a time to death from other causes equal to the seventh decile of time to death among the general female population for that age (i.e., that time by which 70% of 55 year old women would be dead of any cause).

Duration of followup for each patient was selected as a random number uniformly distributed between the limits of the study. For example, in a 5 year study with three years of additional followup, duration of followup for each patient would be a randomly selected line between three and eight years, rounded to a maximum of two decimal places.

For this study, both $\Delta_C$ and $\Delta_M$ were allowed to vary between 0 and 1.0 in increments of 0.1, yielding a total of 33 possible combinations – i.e., $\Delta_C = 0$ to 1.0 while $\Delta_M = 0$, $\Delta_M = 0$ to 1.0 while $\Delta_C = 0$, and $\Delta_C = \Delta_M = 0$ to 1.0. For each combination, a total of 1000 'clinical trials' were 'conducted'. Each set of 33,000 trials was performed with the following parameters:

Set 1: Total patients = 500, additional followup = 3 years
Set 2: Total patients = 500, additional followup = 5 years
Set 3: Total patients = 750, additional followup = 3 years.

For all trials, recruitment occured over 5 years, so that maximum followup was either 8 years (3 years of additional followup) of 10 years (5 years of additional followup).

For each trial, log-rank analysis was performed in the standard fashion (Peto *et al.*, 1977), ending each interval of analysis at the time when one or more deaths occurred.

### Results

Results are shown in Table I. It can be seen that an increase in additional followup from 3 to 5 years enhances the ability of the log-rank test to detect a survival difference produced by an improvement in cured fraction, but diminishes slightly the ability of this method to detect a survival difference produced by enhancement in median survival time. The explanation for this seemingly paradoxical effect can be discovered from an examination of Figures 1a and 1b; with improved cured fraction alone, there is a progressive increase in the survival difference between treated and control patients, while with enhanced median survival time alone, this difference declines after approximately 8 years.

**Table I** Number of significant results (per 1000 trials) from treatment of patients with Stage 2 breast cancer recruited over 5 years with additional followup

| *No. Cases* | *Add. yrs. F/U* | *Covar. incr.* | *Proportional increases* | | | | | | | | | | |
|---|---|---|---|---|---|---|---|---|---|---|---|---|---|
| | | | *0.0* | *0.1* | *0.2* | *0.3* | *0.4* | *0.5* | *0.6* | *0.7* | *0.8* | *0.9* | *1.0* |
| | | | | | | | Threshold $P = 0.05$ | | | | | | |
| 500 | 3 | C | 57 | 68 | 123 | 231 | 308 | 449 | 619 | 769 | 865 | 937 | 970 |
| | | exp {M} | 45 | 90 | 220 | 416 | 617 | 780 | 868 | 954 | 978 | 990 | 998 |
| | | Both | 56 | 151 | 442 | 774 | 931 | 988 | 999 | 999 | 1000 | 1000 | 1000 |
| 500 | 5 | C | 43 | 71 | 125 | 282 | 399 | 567 | 741 | 875 | 920 | 979 | 989 |
| | | exp {M} | 49 | 103 | 216 | 384 | 585 | 739 | 854 | 926 | 968 | 993 | 996 |
| | | Both | 42 | 181 | 499 | 819 | 949 | 994 | 999 | 1000 | 1000 | 1000 | 1000 |
| 750 | 3 | C | 50 | 71 | 145 | 278 | 444 | 658 | 774 | 901 | 962 | 992 | 997 |
| | | exp {M} | 42 | 129 | 307 | 580 | 798 | 910 | 973 | 987 | 998 | 1000 | 1000 |
| | | Both | 40 | 207 | 656 | 916 | 991 | 999 | 1000 | 1000 | 1000 | 1000 | 1000 |
| | | | | | | | Threshold $P = 0.01$ | | | | | | |
| 500 | 3 | C | 9 | 18 | 43 | 84 | 152 | 217 | 372 | 537 | 682 | 824 | 900 |
| | | exp {M} | 5 | 22 | 74 | 210 | 389 | 573 | 705 | 841 | 911 | 952 | 985 |
| | | Both | 11 | 50 | 234 | 550 | 813 | 943 | 992 | 998 | 1000 | 1000 | 1000 |
| 500 | 5 | C | 8 | 17 | 48 | 117 | 208 | 337 | 506 | 701 | 819 | 920 | 973 |
| | | exp {M} | 12 | 25 | 94 | 181 | 357 | 519 | 663 | 802 | 890 | 954 | 979 |
| | | Both | 7 | 68 | 248 | 601 | 824 | 958 | 993 | 1000 | 1000 | 1000 | 1000 |
| 750 | 3 | C | 10 | 16 | 58 | 114 | 213 | 419 | 565 | 768 | 893 | 948 | 981 |
| | | exp {M} | 7 | 36 | 130 | 353 | 586 | 753 | 904 | 966 | 989 | 998 | 999 |
| | | Both | 7 | 90 | 407 | 783 | 955 | 995 | 1000 | 1000 | 1000 | 1000 | 1000 |

### Discussion

The fundamental goal of cancer therapy is to cure patients of their entire tumour burden, so that the treated cancers pose no further threat to life. Unfortunately, given the practical constraints of therapeutic trials, we must rely on a secondary measure of success – improved survival within 5 to 10 years of initial treatment. Such an improvement, as can be seen in Figure 1, does not assure an increase in cure rate, but rather may reflect only an increase in survival time.

Although increased survival time is a worthy goal, its value to the patient must be weighed against the personal and social costs of adjuvant therapy. Women may be less willing to spend months suffering the side-effects of cytotoxic drugs when the potential payoff is months of extra life, rather than a substantial increase in the likelihood of cure. This dichotomy is especially compelling among young women, for whom a cure can mean decades of productive and disease-free life.

Even though several clinical trials have documented the 'benefit' of adjuvant therapy, our analysis offers no reassurance that modern therapy is curing a substantial portion of women with stage 2 breast cancer. On the contrary, a proportional increase in median survival time led to a more consistently detected survival difference by log-rank analysis than an increase of equal proportion in cured fraction. Thus, given the time constraints of many clinical trials, these detectable differences may reflect only a prolongation of survival time.

It is important to note that this limitation is independent of the level of significance achieved with the log-rank test – i.e., an especially small $P$-value does not assure that a treatment benefit results from enhancement of cured fraction. Furthermore, we cannot expect better results from other non-parametric methods of survival analysis (Peto *et al.*, 1977). As this article points out, the log-rank statistic is an excellent test of the null hypothesis (no significant difference in survival at specific endpoint), even if the hazards are not proportional in the two treatment groups, as they were not in our simulated populations. On the other hand, we can detect a specific therapeutic enhancement of cured fraction with certain parametric methods. Unfortunately, these methods often require large data sets and prolonged followup (Boag, 1949; Gamel *et al.*, 1990; Mould & Boag, 1975).

In closing, we must consider the limitations of this study. The findings described above are to some extent dependent on the model we selected. Perhaps the true parameters of patients with stage 2 breast carcinoma differ substantially from those parameters programmed into the hypothetical populations studied in this report. Because of such uncertainties, we would not propose basic changes in the design of clinical trials based on this evidence alone.

One conclusion, however, is clearly drawn, as can be seen in Figure 1. With increasing followup, it becomes progressively more likely that a persistent difference in survival is due to enhanced cure rate rather than enhanced survival time alone. Given this fact, and given the social and financial cost of therapeutic trials, perhaps we should continue indefinitely our efforts to obtain followup data on these patients. Important efforts in this direction have been accomplished by the National Cancer Data Base (USA), which represents a nationwide collection of clinical data from selected cancers (Steele *et al.*, 1992) and by the Cancer Registry of Norway. Furthermore, perhaps we should strive for the largest feasible sample size, even if this exceeds the estimates derived from standard tables, since this would also enhance our ability to detect a true change in the cure rate.

This work was supported by a Veterans Administration Merit Review Research Grant Project 001, the Kentucky Lions Eye Foundation, and an unrestricted grant from Research to Prevent Blindness, Inc. (New York, New York). The opinions or assertions contained herein are the private views of the authors and are not to be construed as official or as reflecting the views of the Department of the Army or the Department of Defense.

### References

BOAG, J.W. (1949). Maximum likelihood estimates of the proportion of patients cured by cancer therapy. *J. Royal Stat. Soc.*, **11**, 15–44.

FREEDMAN, L.S. (1982). Tables of the number of patients required in clinical trials using the logrank test. *Stat. Med.*, **1**, 121–129.

GAMEL, J.W., MCLEAN, I.W. & ROSENBERG, S.H. (1990). Proportion cured and mean log survival time as functions of tumour size. *Stat. Med.*, **9**, 999–1006.

HEWLETT-PACKARD. (1985). *Basic 4.0 manual.* Hewlett-Packard Company: Fort Collins, Colorado.

MORGAN, B.J.T. (1984). Particular methods for non-uniform random variables. In *Elements of Simulation*. pp. 77–90. Chapman-Hall: London.

MOULD, R.F. & BOAG, J.W. (1975). A test of several parametric statistical models for estimating success rate in the treatment of carcinoma cervix uteri. *Br. J. Cancer*, **32**, 529–550.

PETO, R., PIKE, M.C., ARMITAGE, P., BRESLOW, N.E., COX, D.R., HOWARD, S.V., MANTEL, N., MCPHERSON, K., PETO, J. & SMITH, P.G. (1976). Design and analysis of randomized clinical trials requiring prolonged observation of each patient: I. Introduction and design. *Br. J. Cancer*, **34**, 585–612.

PETO, R., PIKE, M.C., ARMITAGE, P., BRESLOW, N.E., COX, D.R., HOWARD, S.V., MANTEL, N., MCPHERSON, K., PETO, J. & SMITH, P.G. (1977). Design and analysis of randomized clinical trials requiring prolonged observation of each patient: II. Analysis and examples. *Br. J. Cancer*, **35**, 1–39.

RUTQVIST, L.E., WALLGREN, A. & NILSSON, B. (1984). Is breast cancer a curable disease? A study of 14,731 women with breast cancer from the cancer registry of Norway. *Cancer*, **53**, 1793.

STEELE, G.D., Jr., WINCHESTER, D.P., MENCK, H.R. & MURPHY, G.P. (1992). *National Cancer Data Base: Annual Review of Patient Care*. American Cancer Society, Inc.: Atlanta, Georgia.


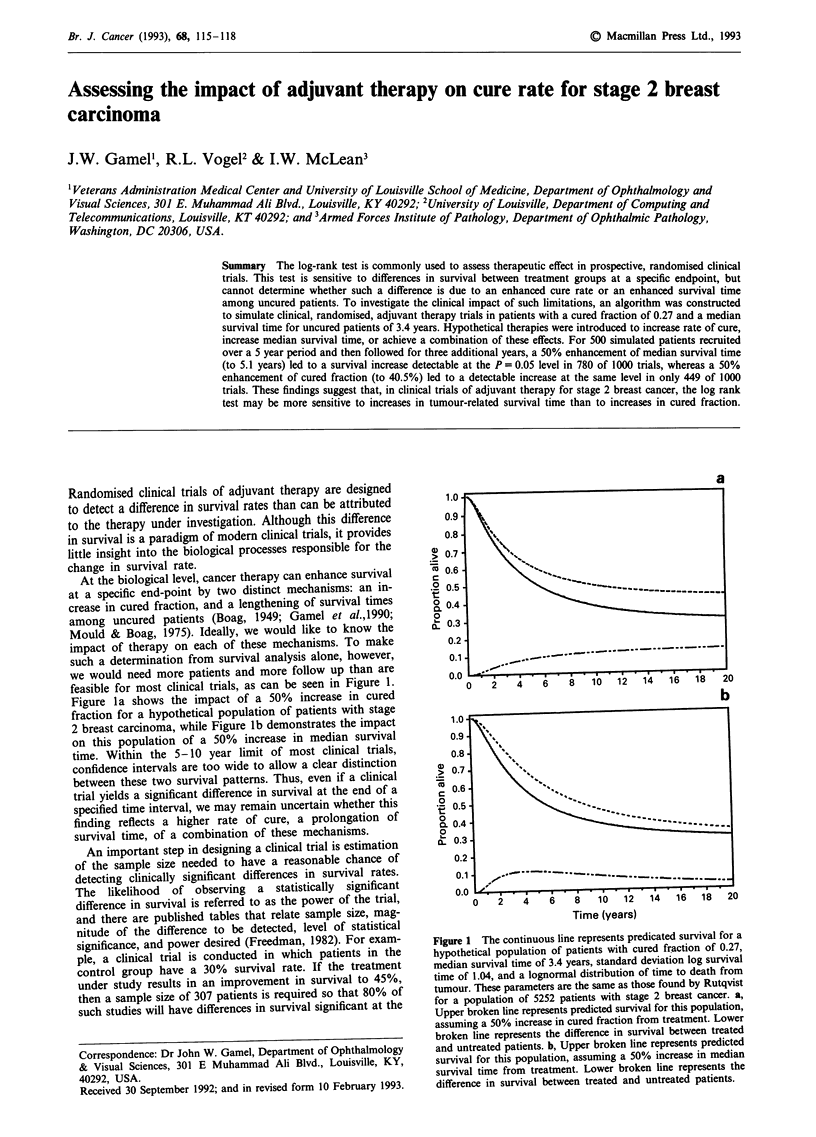

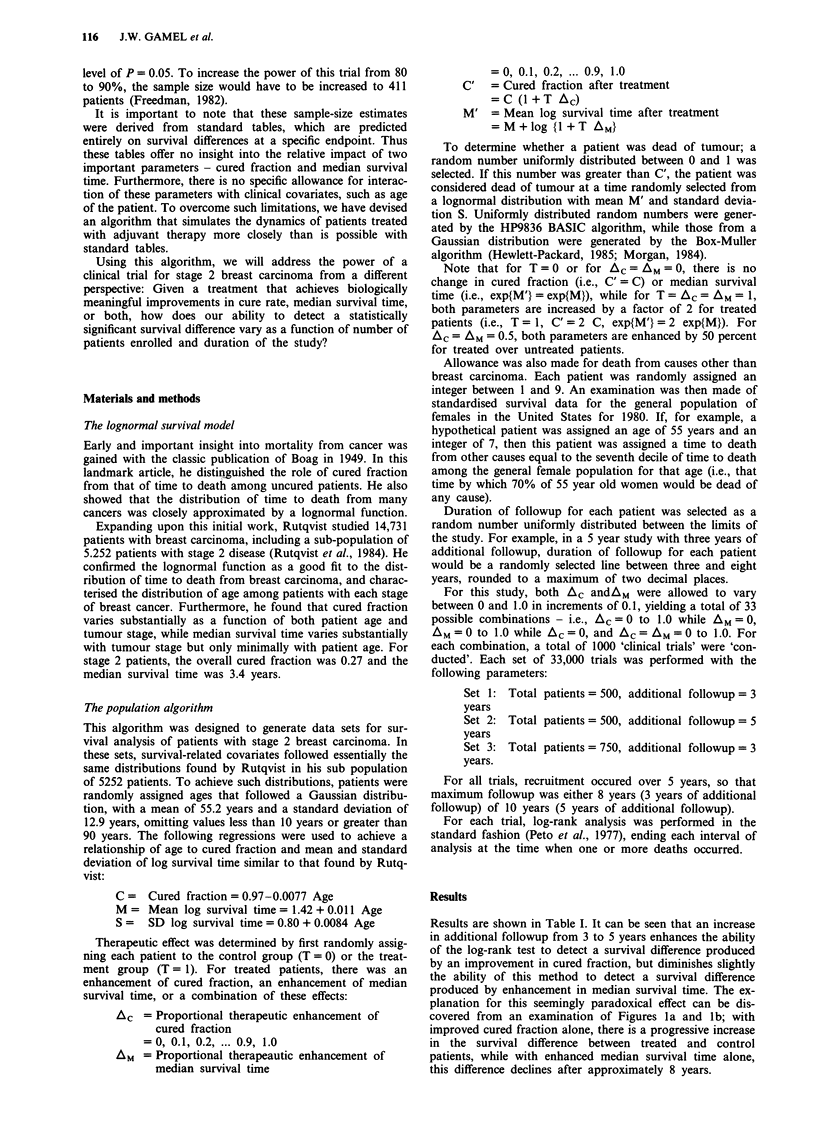

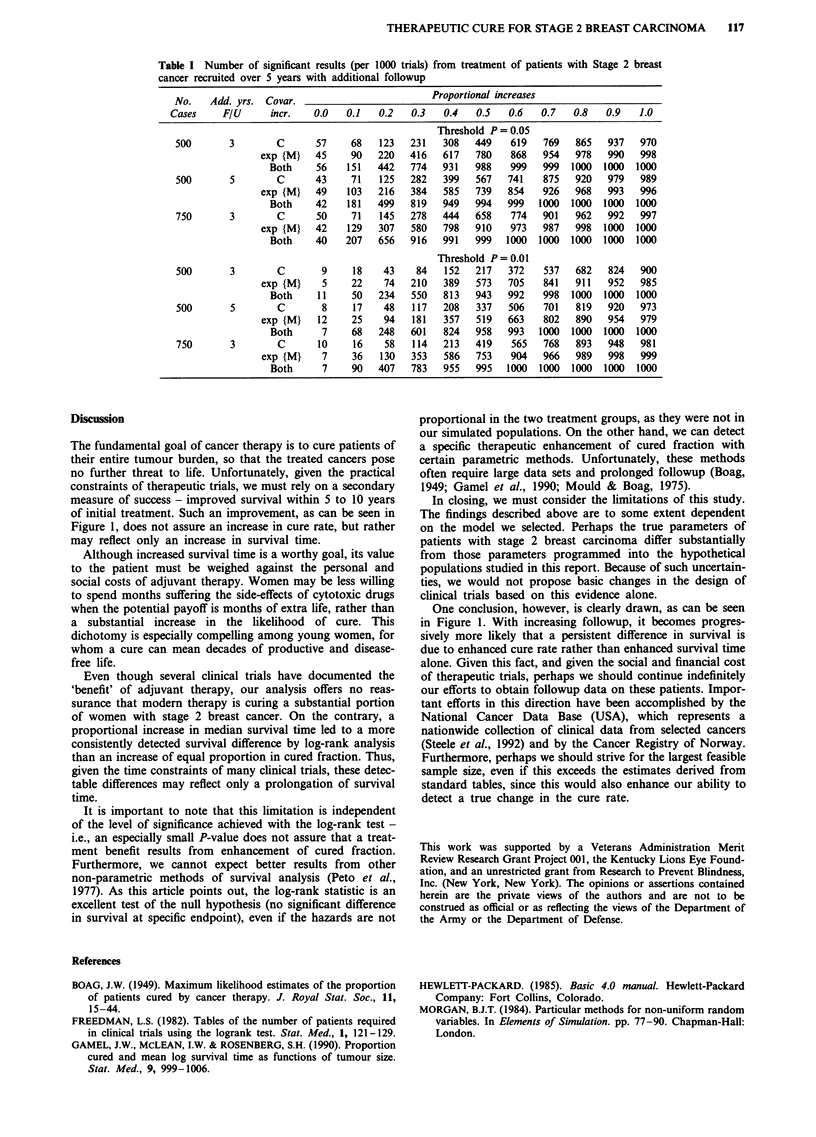

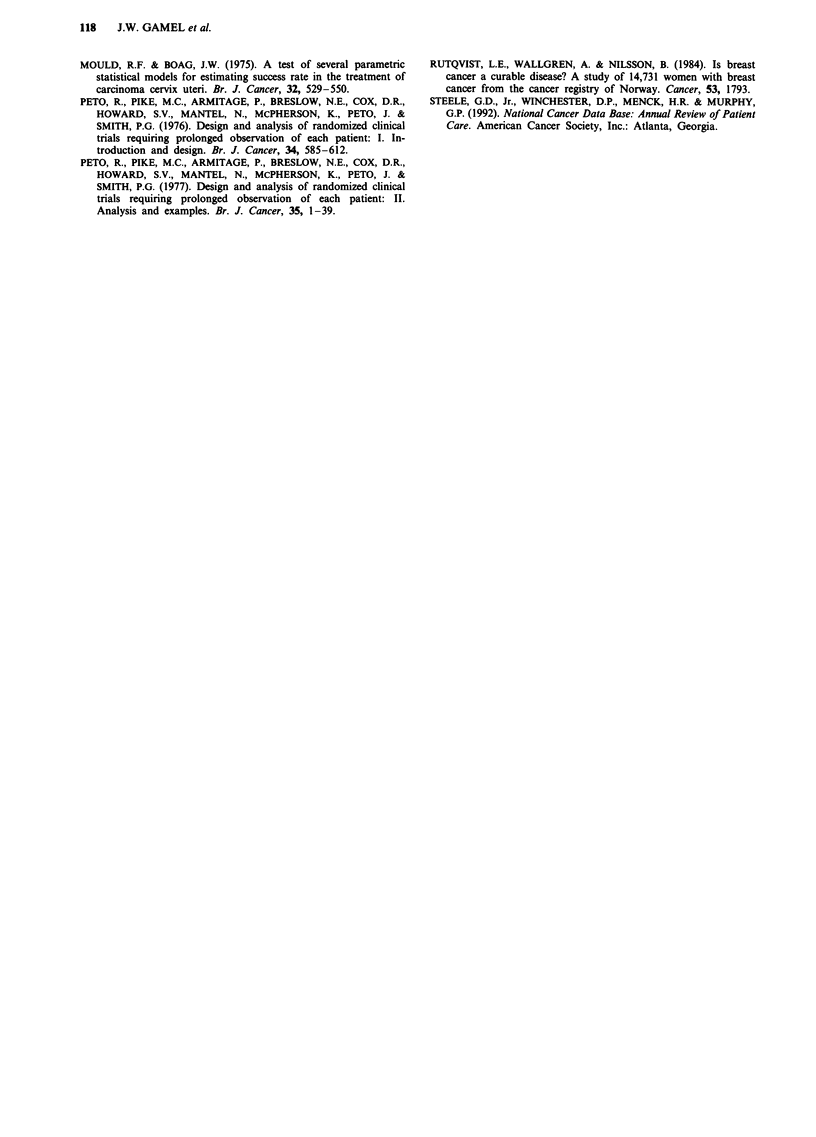


## References

[OCR_00440] Freedman L. S. (1982). Tables of the number of patients required in clinical trials using the logrank test.. Stat Med.

[OCR_00443] Gamel J. W., McLean I. W., Rosenberg S. H. (1990). Proportion cured and mean log survival time as functions of tumour size.. Stat Med.

[OCR_00459] Mould R. F., Boag J. W. (1975). A test of several parametic statistical models for estimating success rate in the treatment of carcinoma cervix uteri.. Br J Cancer.

[OCR_00464] Peto R., Pike M. C., Armitage P., Breslow N. E., Cox D. R., Howard S. V., Mantel N., McPherson K., Peto J., Smith P. G. (1976). Design and analysis of randomized clinical trials requiring prolonged observation of each patient. I. Introduction and design.. Br J Cancer.

[OCR_00471] Peto R., Pike M. C., Armitage P., Breslow N. E., Cox D. R., Howard S. V., Mantel N., McPherson K., Peto J., Smith P. G. (1977). Design and analysis of randomized clinical trials requiring prolonged observation of each patient. II. analysis and examples.. Br J Cancer.

[OCR_00478] Rutqvist L. E., Wallgren A., Nilsson B. (1984). Is breast cancer a curable disease? A study of 14,731 women with breast cancer from the Cancer Registry of Norway.. Cancer.

